# Different Degree in Proteasome Malfunction Has Various Effects on Root Growth Possibly through Preventing Cell Division and Promoting Autophagic Vacuolization

**DOI:** 10.1371/journal.pone.0045673

**Published:** 2012-09-21

**Authors:** Xianyong Sheng, Qian Wei, Liping Jiang, Xue Li, Yuan Gao, Li Wang

**Affiliations:** 1 College of Life Sciences, Capital Normal University, Beijing, China; 2 Key Laboratory of Molecular Physiology, Institute of Botany, The Chinese Academy of Sciences, Beijing, China; 3 School of Biological Science and Technology, Central South University, Changsha, China; University of Michigan, United States of America

## Abstract

The ubiquitin/proteasome pathway plays a vital role in plant development. But the effects of proteasome malfunction on root growth, and the mechanism underlying this involvement remains unclear. In the present study, the effects of proteasome inhibitors on Arabidopsis root growth were studied through the analysis of the root length, and meristem size and cell length in maturation zone using FM4–64, and cell-division potential using GFP fusion cyclin B, and accumulation of ubiquitinated proteins using immunofluorescence labeling, and autophagy activity using LysoTracker and MDC. The results indicated that lower concentration of proteasome inhibitors promoted root growth, whereas higher concentration of inhibitors had the opposite effects. The accumulation of cyclin B was linked to MG132-induced decline in meristem size, indicating that proteasome malfunction prevented cell division. Besides, MG132-induced accumulation of the ubiquitinated proteins was associated with the increasing fluorescence signal of LysoTracker and MDC in the elongation zone, revealing a link between the activation of autophagy and proteasome malfunction. These results suggest that weak proteasome malfunction activates moderate autophagy and promotes cell elongation, which compensates the inhibitor-induced reduction of cell division, resulting in long roots. Whereas strong proteasome malfunction induces severe autophagy and disturbs cell elongation, resulting in short roots.

## Introduction

Root is a very important organ growing downward into the soil to anchor the plant as well as take up water and mineral ions. Root growth is determined by the meristem cell division and subsequently cell elongation-differentiation [1]. The control of this type of growth requires a number of factors and activities to be integrated in space and time. The ubiquitin/proteasome pathway (UPP) is one of the most important proteolytic pathways in eukaryotic cells involving in the degradation of the bulk of intracellular proteins, including misfolded proteins and short- and long-lived regulatory proteins [2]–[4]. It was reported that seed formation and germination was accompanied by the dynamic changes of the ubiquitinated proteins in root [5]. Besides, the mass distribution of both ubiquitinated proteins and proteasome were also observed in the root and shoot apical meristems [6]. All these data indicated that UPP was playing a role in regulating the root growth.

However, the data available at present appears insufficient to provide complete knowledge of the functions of the UPP during root development. For example, analysis of several proteasome mutations with different degree in proteasome malfunction indicated that the majority of the UPP mutants have shorter root [7]–[9]. On the other hand, a more recent study indicated that down-regulation of the UPP activity by application of proteasome inhibitor stimulated the *shr* root elongation [10]. Obviously, the effects of proteasome malfunction on the root growth during postgermination development are still a debatable question. Furthermore, several researches indicated that slight loss of proteasome function leaded to an enlargement of cell size in leaves, stems, flowers, fruits, seeds, and embryos [7], [8], [11], [12]. But so far we know very little about the mechanism underlying this phenomenon. Particularly, no attention has been paid to the possible activation of autophagic vacuolization in response to proteasome malfunction [13], [14], which are closely linked to cell enlargement [15].

To extend our knowledge of the involvement of the UPP in root growth, we provide here several lines of evidence about effects of the peptide aldehyde proteasome inhibitor on Arabidopsis root growth, providing further insights into the mechanism by which the UPP controls plant growth.

## Materials and Methods

### Chemicals

MG132, MG115, E-64, and Dansylcadaverine (MDC) were purchased from Sigma. LysoTracker Green and FM4–64 were purchased from Invitrogen. The stock solutions of MG132 (40 mM), MG115 (40 mM), FM4–64 (500 µg ml^−1^) and LysoTracker Green (1 mM) were prepared using dimethylsulfoxide (DMSO) as solvent. E-64 (20 mM) and MDC (25 mM) were dissolved in water.

### Growth conditions

Surface sterilized seeds of *Arabidopsis thaliana* (Col-0) were cold-pretreated at 4°C for 48 h, and cultured in 1/2 MS with 1% Suc for 24 h, and then uniform seedlings of similar size and primary root length were transferred to the same fresh medium containing different proteasome inhibitors MG132, MG115, and Cys-protease inhibitor E-64, for additional 48 h. Each proteasome inhibitors were used at concentrations of 0, 20, 40, and 80 µM. DMSO controls were set up by adding the similar amount of DMSO solvent.

### Measurement of root length

Images of seedlings were acquired with a Zeiss Stemi SV11 stereoscopic microscope (Germany), equipped with an AxioCam HRc digital camera, and the lengths of the primary roots were measured using AxioVision Rel 4.8. The experiment was repeated thrice with consistent results. The values were expressed as the means ± SD for 15 seedlings, of each treatment.

### FM4–64 staining

Roots of both control and treated were stained with FM4–64 (2.5 µg ml^−1^) for 2 min, and then visualized using 40× objective of a Zeiss 5 live confocal microscope equipped with an automated microscope stage and a 561 nm laser (Germany). Images were recorded and analyzed with the Zeiss Zen2007 software.

### Immunocytochemical analysis of ubiquitinated proteins

Samples for immunocytochemical analysis were prepared as similar to earlier works [16], [17]. Briefly, samples were fixed and embedded in LR White resin (Sigma). Semi-thin sections (1 µm) were blocked with 3% BSA, and then incubated with an anti-ubiquitin antibody (1∶8; Sigma) and then followed by FITC-conjugated IgG (1∶10; Boisynthesis, Beijing, China). All the samples were examined under the ZEISS laser scanning confocal microscope equipped with a 488 nm laser (Germany). All the sections were incubated simultaneously and using the similar antibodies, except for the negative controls that were treated as described, but omitting the primary antibody. Laser power and channel setting for FITC were kept identical for all samples to make the results comparable.

### Localization of cyclin B

Arabidopsis with GFP-fusion cyclin B (kindly provided by associated Prof. Bai from CNU) was treated with various concentrations of MG 132. All seedlings were stained with FM 4–64 (2.5 µg ml^−1^), and then examined under the ZEISS 5 live laser scanning confocal microscope (Germany), with a 488 nm laser for GFP, and a 561 nm laser for FM 4–64. To make the results comparable, laser power and channel setting for GFP were kept identical for all samples.

### LysoTracker or MDC staining

LysoTracker or MDC staining were performed according to earlier studies [18], with some modification. Roots (both control and MG132-treated) were initially incubated with LysoTracker Green (2 µM) for 25 min, or with MDC (125 µM) for 2 min. They were then washed with 1/2 MS,and incubated with FM4–64 (2.5 µg ml^−1^) for 2 min. All samples were visualized under the confocal microscope, with a 405 nm laser for MDC, with a 488 nm laser for LysoTracker –Green, and with a 561 nm laser for FM 4–64. To make the results comparable, laser power and channel setting for MDC and LysoTracker-Green were kept identical for all samples.

### Statistics

One-way ANOVA was used to compare the difference between the treated and control pollen tubes. Values of P<0.05 were taken as statistically significant.

## Results

### Proteasome malfunction has various effects on primary root growth

**Figure 1:**
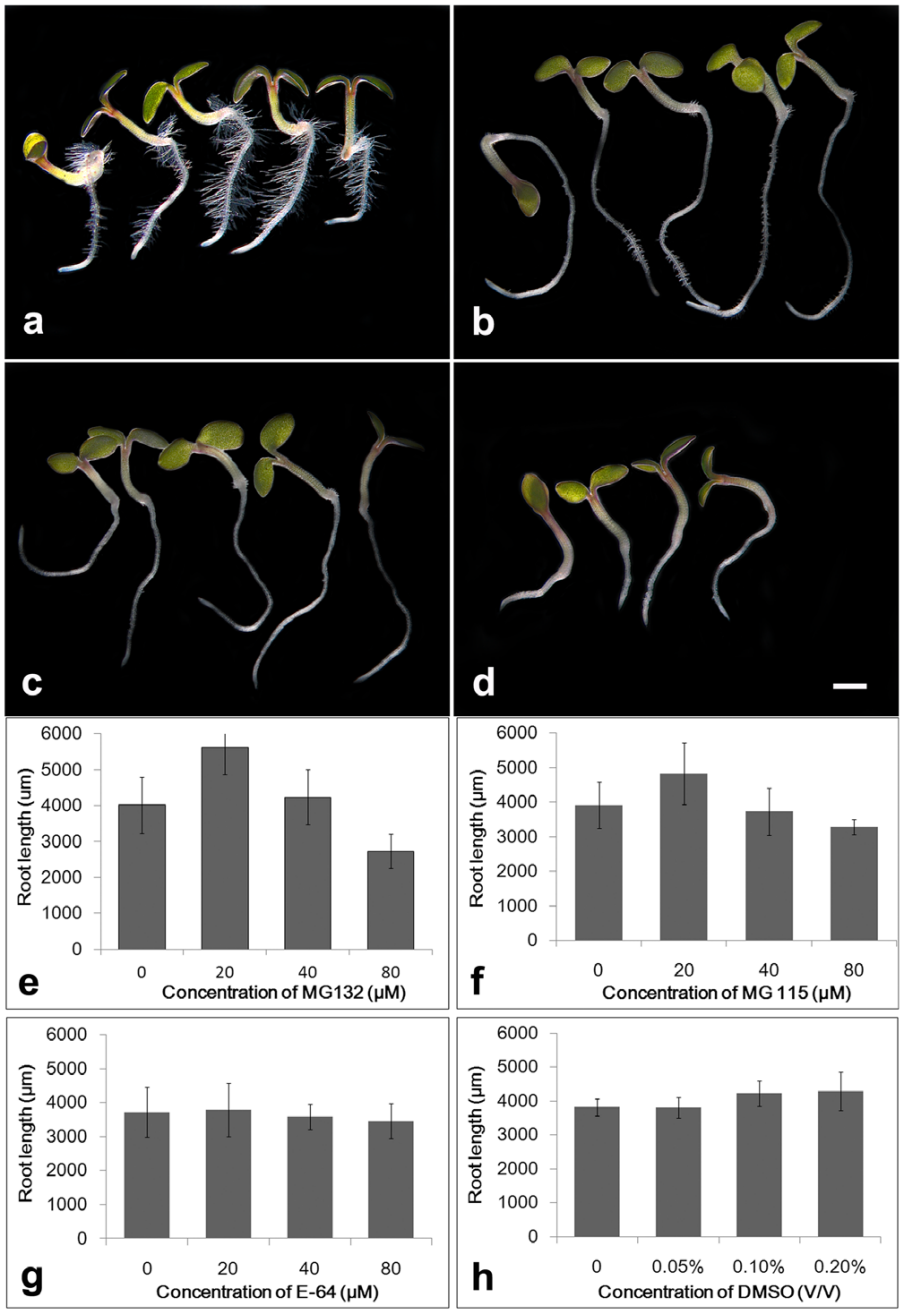
Effects of different inhibitors on primary root elongation. a–d. Cold-pretreated seeds were cultured in 1/2 MS with 1% Suc for 24 h, and then uniform seedlings of similar size and primary root length were transferred to the same fresh medium containing 0 (a), 20 µM MG132 (b), 40 µM MG132 (c) or 80 µM MG132 (d), respectively, for additional 48 h. Bars = 1000 µm. e–h. Cold-pretreated seeds were cultured in 1/2 MS with 1% Suc for 24 h, and then uniform seedlings of similar size and primary root length were transferred to the same fresh medium containing various concentrations of MG 132 (e), MG115 (f), E-64 (g) and DMSO (h), for additional 48 days. The values are the means ± SD of about 15 seedlings of each treatment. The experiment was repeated thrice with consistent results.

To assess the roles of the UPP during root growth, seeds of Arabidopsis were cultured under standard conditions for 24 h, and then seedlings with homologous root length were treated with 0, 20, 40 or 80 µM MG132 for additional 48 h, respectively. Microscopic analysis revealed that MG132 treatment has various effects on Arabidopsis root growth in a dose-dependent manner. The seedlings cultured under control conditions for 72 h showed normal morphology with an average length of 4011±785 µm (Fig. 1 a, e). In contrast, the data of seedlings treated with concentrations of MG132 were 5629±764 µm (20 µM), 4233±766 µm (40 µM), or 2731±476 µm (80 µM) (Fig. 1 b–e). In other words, the root length of seedling treated with MG132 was approximately 40.3% longer (20 µM), or 31.9% shorter (80 µM), when compared with untreated control (P<0.05). Similar phenomena were observed when MG115, another tripeptide aldehyde, was used (Fig. 1 f, data not shown).

Given that tripeptide aldehydes can also inhibit calpains as well as proteasome [16], [19], the effects of E-64 as a Cys-protease inhibitor, were also investigated in our experiments. The results indicated that though 80 µM E-64 slightly inhibited root elongation, no significant differences were observed in root lengths between control and low concentration of E-64 treatment (Fig. 1 g, data not shown), indicating that effects of both MG132 and MG115 on root growth were not due to the inhibition of other protease activities. Besides, since both MG 132 and MG 115 were dissolved in DMSO, part of samples was treated with same concentration of DMSO without tripeptide aldehyde. The obtained results indicated that 0.1–0.2%, but not 0.05%, DMSO slightly promoted root growth (Fig. 1 h, data not shown), thus excluding the possible effects of DMSO on root growth.

### MG132 reduces meristem size through declined cell division and accelerated cell differentiation

**Figure 2:**
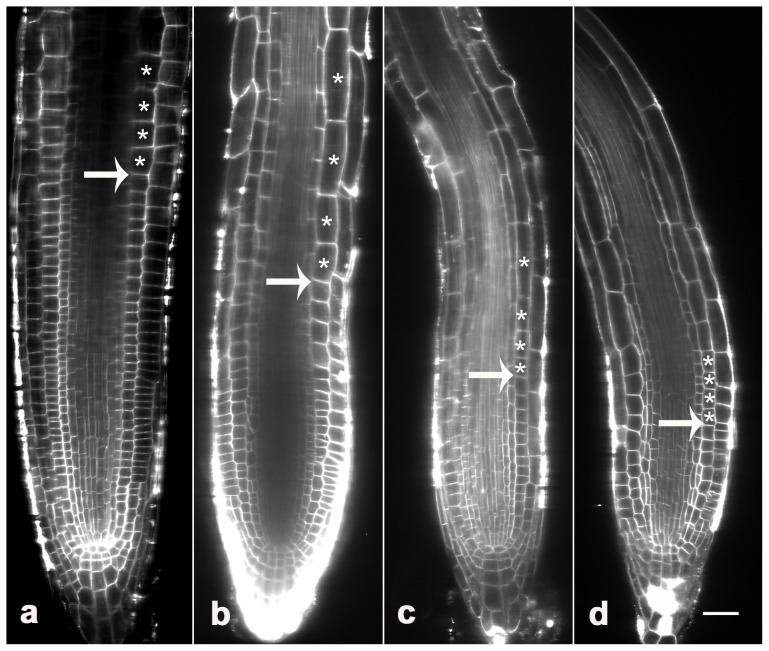
Effects of MG132 on root-meristem size. Roots treated with 0 (a), 20 µM MG132 (b), 40 µM MG132 (c) or 80 µM MG132 (d) were stained with FM 4–64, and longitudinal views were obtained using a Zeiss confocal. The results show reduced root-meristem size (indicated by arrows) and accelerated cell differentiation (indicated by stars) in response to MG132 treatment. Bars = 20 µm.

Root growth is sustained by the root meristem [20]. To clarify the mechanism by which proteasome controls root growth, we analyzed the effects of MG132 on meristem size by labeling whole-mount seedlings with FM4–64 [21]. The results indicated that the average meristem cell number of cortex cells in a file extending from the quiescent center to the first elongated cell [1], in 3-d untreated seedlings was about 32±2, with a total length of about 281±15 µm (Fig. 2 a). By contrast, the cell number and length of meristem in MG132-treated seedlings were 28±4/240±15 µm (20 µM), 25±3/201±31 µm (40 µM), and 13±2/144±12 (80 µM) (Fig. 2 b–d). All these data indicated that MG132 treatment caused a significantly decrease in meristem size in a dose-dependent manner.

**Figure 3:**
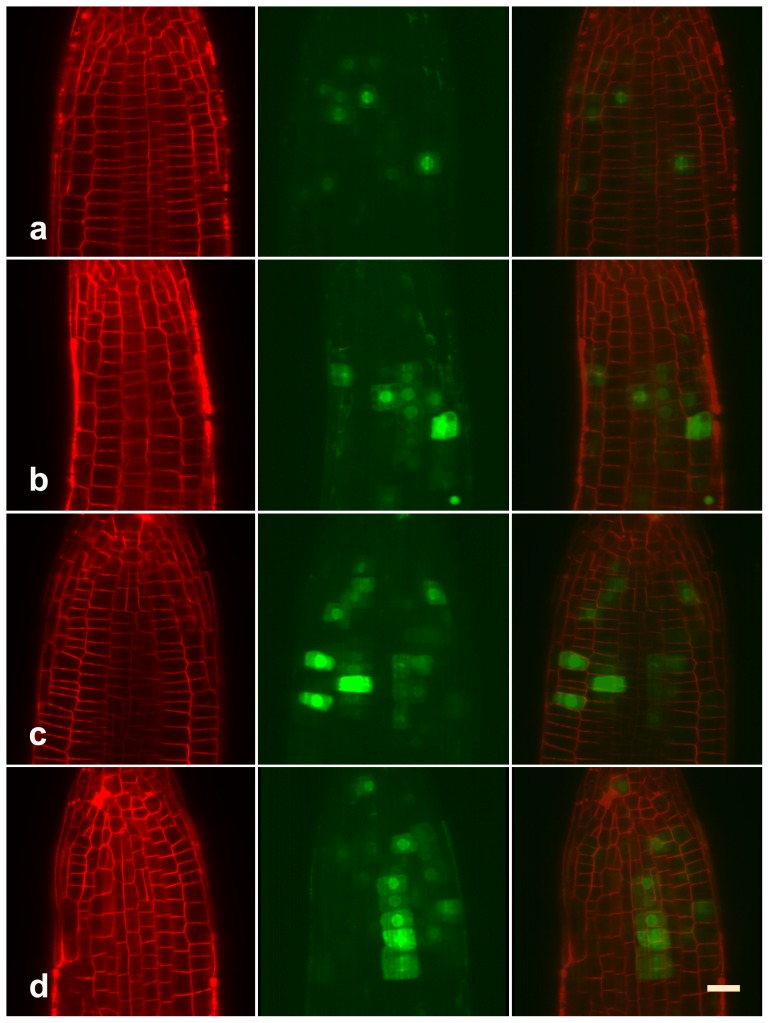
GFP-fusion cyclin B analysis. Arabidopsis carrying GFP-fusion cyclin B were treated with 0 (a), 20 µM (b), 40 µM (c), or 80 µM (d) MG132, respectively. Longitudinal views of the GFP (left) and FM 4–64 (middle) fluorescent emissions were collected separately and then merged (right), using a Zeiss confocal. Figures show accumulation of cyclin B in root-meristemic zone in response to MG132 treatment. Bar = 20 µm.

Besides, FM staining also revealed that MG132 treatment is concomitant with a more rapid cell differentiation in the elongation zone (Fig. 2). Given that root-meristem size is determined not only by the rate of cell differentiation, but also by the rate of cell division [1]. To assess whether the decline in root meristem size could also be caused by reduce of cell-division potential, we analyzed the turnover of cyclin B, an essential protein expressed during the G2 phase of the cell cycle, and degraded during mitosis [22], [23]. Confocal microscopic analysis revealed that untreated Arabidopsis showed some GFP-fusion cyclin B spotted in their roots (Fig. 3 a), showing cells in M phase [24]. In contrast, both the percentage of GFP-labeled cells and the relative intensity of fluorescent signal slightly increased in roots treated with 20 µM MG132 (Fig. 3 b). And far more cyclin B signals were detected in roots treated with higher concentration of MG132 (Fig. 3 c–d). These data suggested that any level of proteasome malfunction would disturb the degradation of cyclin B, resulting in the abnormal mitosis in root meristem.

### Proteasome malfunction promotes, or prevents root cell elongation

**Figure 4:**
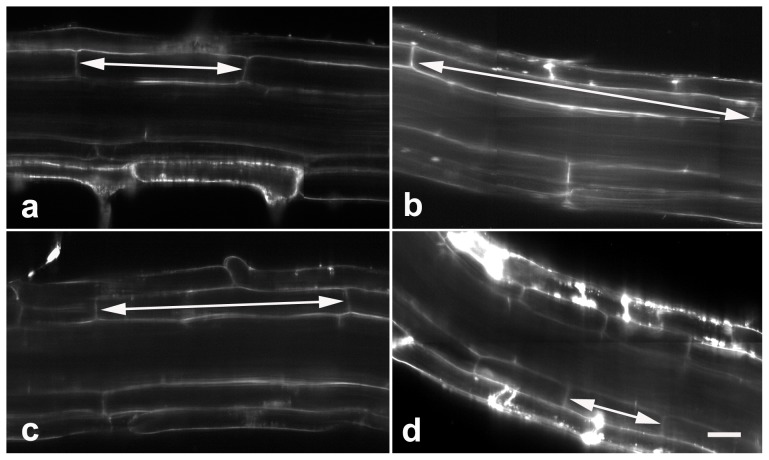
MG132 alters cell length in the maturation zone. Roots treated with 0 (a), 20 µM MG132 (b), 40 µM MG132 (c) or 80 µM MG132 (d) were stained with FM 4–64, and longitudinal views were obtained using a Zeiss confocal. The results show that MG132 alters cell length in the maturation zone (indicated by arrows). Bars = 20 µm.

The final size of an organ depends on the number and volume of its cells [25]. Since proteasome malfunction disturbed cell division in the meristem zone, we therefore speculated that different concentration of proteasome inhibitors might have various effects on the cell elongation. To validate this possibility, analysis on the length of cortex cell in the maturation zone was performed. Microscopic evaluation revealed that the average cell length of untreated seedlings was about 116±21 µm (Fig. 4 a). By contrast, the data of 20 µM MG132-treated seedlings was about 187±23 µm (Fig. 4 b), which was approximately 61.9% longer when compared with untreated control. Though the average cell length of 40 µM MG132-treated seedlings was 143±44 µm (Fig. 4 c), approximately 23.9% longer than the data of control, it was obviously shorter compared with 20 µM MG132-treated ones. On the other hand, the data of seedlings treated with 80 µM MG132 was only 87±31 µm (Fig. 4 d), approximately 24.6% shorter than untreated control. These data indicated that MG132 might promote, or prevent cell elongation, depending on the level of proteasome malfunction.

### MG132-induced accumulation of ubiquitinated proteins is associated with autophagic vacuolization

**Figure 5:**
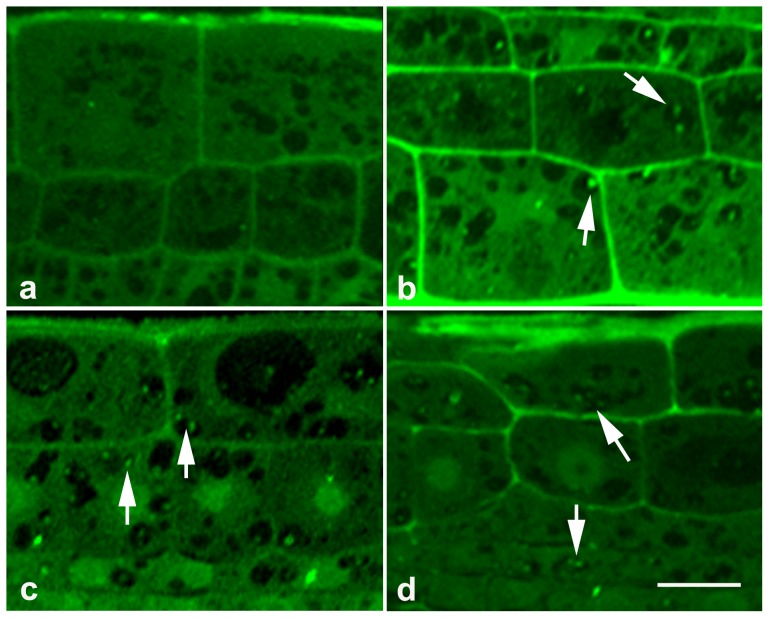
Immunofluorescence labeling of ubiquitinated proteins in root cells. Semi-thin sections of roots treated with 0 (a), 20 µM MG132 (b), 40 µM MG132 (c) or 80 µM MG132 (d) were incubated with anti-ubiquitin antibody and FITC-IgG, and examined with a Zeiss confocal. Arrows show the MG132-induced accumulation of ubiquitinated proteins in vacuoles. Bar = 20 µm.

In the present study, we also found that MG132 treatment is concomitant with strongly cytoplasmic vacuolization in the elongation zone (Data not shown). Given that most substrate proteins are usually ubiquitinated before being degraded by the proteasome [2]–[4], and that inhibition of proteasome activity induces toxic accumulation of ubiquitinated proteins [16]. We therefore speculated that MG132-induced toxic accumulation of ubiquitinated proteins might be associated with the formation of cellular vacuolization in root cells. To validate this speculation, immunofluorescence analysis was carried out using anti-ubiquitin antibody. The obtained results revealed that detectable fluorescent signal was seen in both nuclei and cytoplasm in untreated root cells (Fig. 5 a). On the other hand, a far brighter fluorescence was detected in root treated with 20 µM MG132 (Fig. 5 b). Particularly, strong dot-pattern antigen signal were observed in almost every vacuoles, which were rarely observed in untreated roots (Fig. 5 a). And the increase in the intensity of fluorescence became more obvious in roots treated with 40 µM MG132 (Fig. 5 c). But the magnitude of fluorescent intensity was not significantly higher in root treated with 80 µM MG132 than in that treated with 40 µM MG132 (Fig. 5 d).

**Figure 6:**
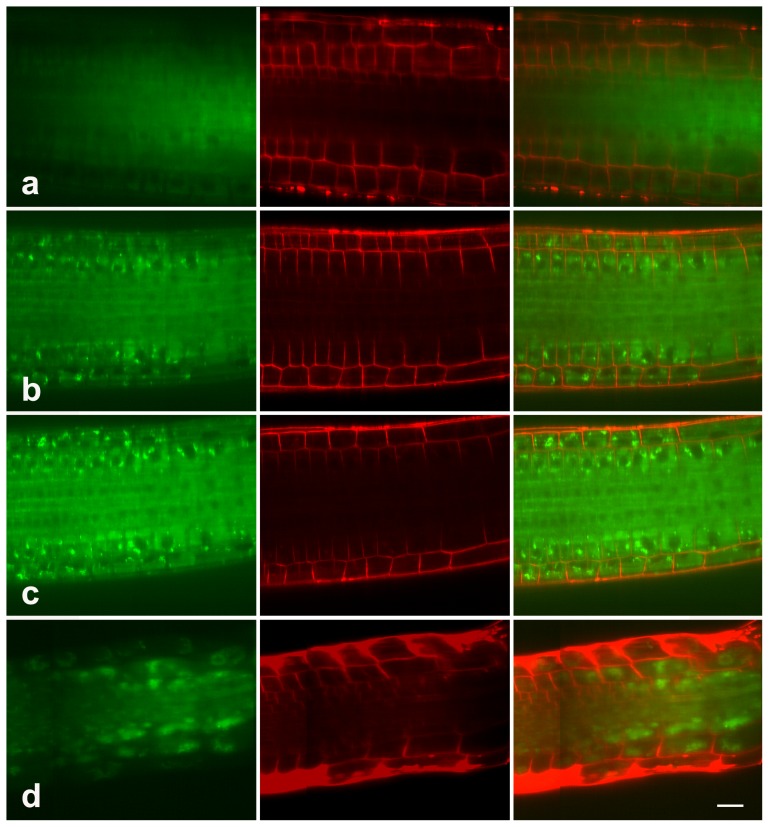
LysoTracker staining in root cells. Seedlings treated with 0 (a), 20 µM (b), 40 µM (c), or 80 µM (d) MG132 were stained with LysoTracker (Green) and FM 4–64 (Red), and examined with a Zeiss confocal. The data show the accumulation of LysoTracker in root cells in response to MG132 treatment. Bar = 50 µm.

Previous reports on animal system have indicated a crosstalk between the UPP and lysosome/vacuole-dependent autophagy [14], [26]. We therefore further speculated that proteasome malfunction might also activate autophagy in root cells. To verify this possibility, we initially stained the untreated and MG132-treated roots with Lysotracker-Green, a fluorescent acidotropic probe widely used to visualize acidic compartments such as lysosome/vacuole [27]. The obtained results revealed that a faint fluorescent signal could be detected in untreated root, mainly in the elongation zone, but not in the meristem zone (Fig. 6 a). In contrast, LysoTracker-stained structures were obviously observed in 20 µM MG132-treated roots, in both the meristem zone and elongation zone (Fig. 6 b). And the fluorescent intensity becomes far stronger as the concentration of MG132 increases to 40 µM (Fig. 6 c). Larger LysoTracker-stained structures were usually observed in 80 µM MG132-treated roots, the magnitude of fluorescent intensity was not significantly higher in 80 µM MG132- treated roots than that in 40 µM MG132-treated ones (Fig. 6 d).

**Figure 7:**
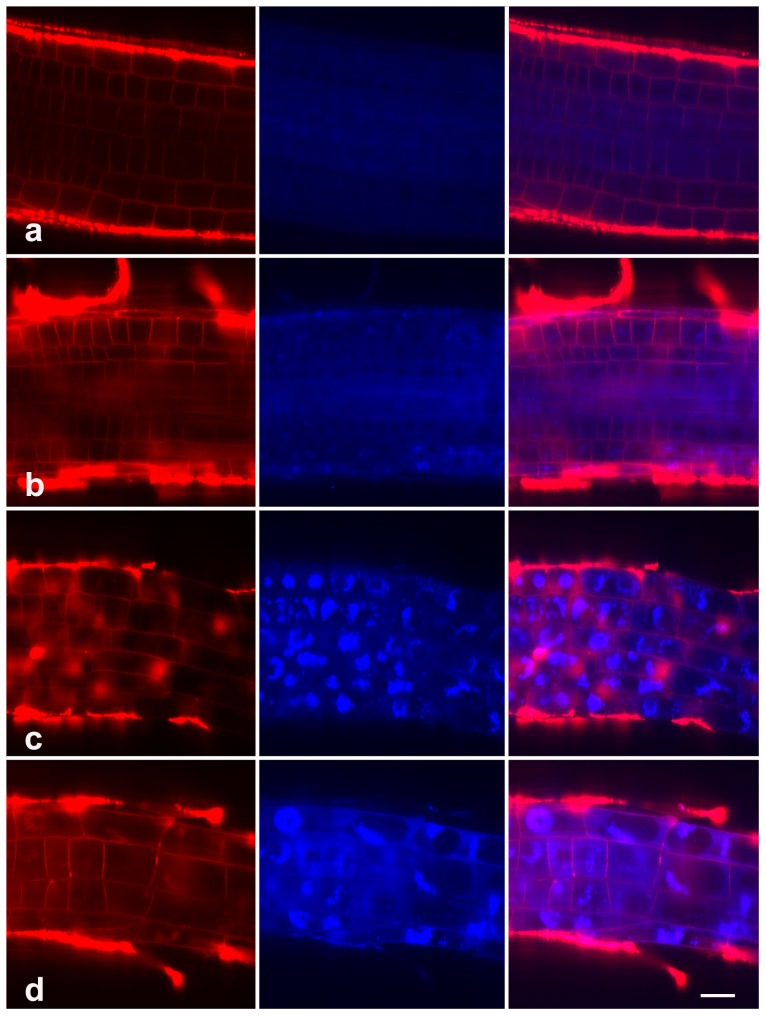
MDC staining in root cells. Seedlings treated with 0 (a), 20 µM (b), 40 µM (c), or 80 µM (d) MG132 were stained with MDC (Blue) and FM 4–64 (Red) and examined with a Zeiss confocal. The data show the accumulation of MDC in root cells in response to MG132 treatment. Bar = 20 µm.

Though the ability of LysoTracker to label autophagy in plants has been demonstrated in various organisms including Arabidopsis [18], [28], [29], it was believed that the dye must be used in combination with other markers of autophagy in order to discriminate autophagic activity from other events increasing lysosome/vacuole activity [27]. We thus used MDC, a commonly used autophagic dye, as a more specific marker to detect autophagy [27], [30]. The results indicated that few MDC-stained structures were visible in the elongation zone of untreated root (Fig. 7 a). In contrast, 20 µM MG132-treated cells contained numerous cytoplasmic structures that concentrated MDC (Fig. 7 b) that were similar in appearance to the starvation-induced autolysosomes in BY-2 cells [30]. And far more MDC-stained structures were observed in 40 µM MG132 treated roots (Fig. 7 c). Strikingly, the amounts of MDC-stained structures seemed to be the highest in 80 µM MG132-treated roots (Fig. 7 d). These data demonstrated the dose-dependent activation of autophagy in response to proteasome malfunction in roots.

## Discussion

Application of proteasome inhibitors is one of the main strategies to study the role of the UPP during plant development. In the present study, we utilized MG132 and MG115, two widely used proteasome inhibitors in both plant and animal systems, to determine the effects of proteasome malfunction on primary root development. The obtained results indicated that different concentration of MG132 or MG115 had diverse effects on root growth (Fig. 1). Nevertheless, the peptide aldehyde may inhibit proteasome as well as calpains [16], [19]. The facts that both MG132 and MG115 showed similarly effects on root development, and that no significant differences were observed in E-64 or DMSO-treated seedlings, enabled us to confirm that the diverse effects on root growth in response to MG132 or MG115 treatment were mainly due to the inhibition of proteasome activity.

Previous reports indicated that weak loss of proteasome function displayed enlarged plant organs, including cotyledons, leaves, petals, shoots and matured seeds, [7], [8], [12]. On the other hand, defective in proteasome activity seemly inevitably showed shorter roots [8], [9], [31], with the only exception that proteasome malfunction stimulated the elongation of *shr* roots [10]. In the present study, our data revealed that lower concentration of MG132 or MG115 markedly promoted Arabidopsis root elongation, while higher concentration of the inhibitors, on the contrary, significantly inhibited root elongation (Fig. 1). Proteasome abundance and activity vary spontaneously during plant development and in response to changes in environmental conditions [11], [17], [32]. So we hypothesized that fluctuations in total proteasome activity might be an important mechanism for controlling the sizes of plant organs, including roots.

The rate of root growth is determined by the rate of the cell division in the meristematic zone and cell differentiation in the elongation zone [1], [20], [25]. The control of these processes requires a number of factors and activities to be integrated in space and time. Previous reports have indicated that several proteins known to be required for the meristematic activity are believed to be regulated by the UPP [23], [33]. Furthermore, it was reported that mutation in 26S proteasome subunit RPT 2a resulted in the loss of the QC identity, as well as the cellular organization and normal nature of the RAM in Arabidopsis [9]. In the present study, our data indicated that MG132 significantly reduced the meristem size in a dose-dependent manner (Fig. 2). Moreover, confocal analysis revealed the positive relation between the accumulation of cyclin B and MG132 treatment (Fig. 3). These data are consistent and confirm the theoretical predication that proteasome-dependent proteolysis is required to control the meristematic activity, and that any level of proteasome malfunction would result in the loss of cell division potential in the root.

The final sizes of determinate organs are believed to be controlled not only by cell number and size, but also by compensation, a still largely unclear mechanism that leads to the enlargement in cell size in an organ with reduced cell number [34], [35]. In fact, proteasome mutation induced compensation in matured seeds, cotyledons, leaves, and petals have been observed [7], [8], [12]. But compensation was not believed to occur in indeterminate organs such as root [7], [34]. In the present research, our new findings revealed that low concentration of MG132 markedly promoted cell elongation, resulting in the far longer longitudinal length of cells in the maturation zone (Fig. 4), which compensated MG132-induced decline in cell number. We therefore hypothesized that compensation might be a ubiquitous phenomenon occurring in all aspects of plant organ development, including root. Also, our data revealed that higher concentration of MG132 severely inhibited cell division, comparing with slightly increase or even disruption in cell elongation (Fig. 4). These effects of MG132 enabled us to propose that MG132-induced compensation tends to be observed only when proteasome activity still exceeds a critical threshold. And we might further hypothesize that the critical threshold of proteasome activity might be organ specific, which has indeed been reported by a previous research that all proteasome mutants tested showed larger petals than those of the wild type, but the rosette leaves were larger only in the *rpt 2a* and smaller in *rpn10* and *rpn12a* [7].

Cell enlargement is often associated with endoreplication and/or vacuolization expansion [15]. Though the UPP defection induced endoreduplication has been widely reported [8], [36], the fact that cell and organ enlargement was not correlated with increased endoreduplication [8], indicating that the general increase in cell expansion in the UPP mutants was not driven by increased polyploidization. On the other hand, proteasome malfunction inevitably induced cytosolic vacuolization in a dose- and time-dependent manner in cells of both animal and plant systems [16], [37]–[40], though the mechanism of such phenomena is still unclear.

The lysosomal/vacuole dependent autophagy is another main protein degradation system characterized by the formation of autophagic vacuoles and fusing with lysosomes/vacuole to form autophagolysosomes, where proteins are degraded [41]. In the present study, immunofluorescence analysis revealed the accumulation of the ubiquitinated proteins, particularly in the vacuoles, in response to proteasome malfunction (Fig. 5). Besides, fluorescent staining with LysoTracker Green and MDC showed that MG132 induced dramatic autophagy in a dose-dependent manner (Figs. 6–7). All these data have enabled us to speculate that MG132-induced accumulation of ubiquitinated proteins might be associated with autophagic vacuolization as a compensatory pathway for proteolysis, which was indeed observed in the earlier investigations on animal cells. For example, Ding et al [13] revealed that autophagy was likely activated in response to endoplasmic reticulum stress caused by accumulation of ubiquitinated proteins during proteasome malfunction in DU145 cell lines. More recently, Du et al [14] reported the activation of autophagy in response to proteasome inhibitor-induced neurotoxicity.

More interestingly, our data indicated that lower concentration of MG132 induced moderate activation of autophagy, associated with promoted cell elongation; while higher concentration of MG132 induced vigorous activation of autophagy, concomitant with disrupted cell elongation. Yet, we still cannot explain the precise mechanism leading to this phenomenon. The most probable explanation is that slight activation of autophagy was capable of protecting the cells through degrading damaged organelles and misfolded proteins, while vigorous activation of autophagy was harmful to cells due to an excessive elimination [14], [42]–[44]. Besides, considering the pleiotropic impact of proteasome inhibition on plant growth, other explanations remain possible. For example, proteasome malfunction might also affect hormone response [31], [45], cytoskeleton organization [16], [46], and the deposition of cell wall components [16], all of which are closely linked to plant cell development.

In summary, our investigation revealed that inhibition of proteasome not only reduced the cell division, but also induced autophagic vacuolization in a dose-dependent manner, resulting in promoted or prevented root elongation. Our data provides providing a further insight into the mechanism by which proteolysis controls root growth.
